# The Thyroid Gland, in Health and Disease

**Published:** 1919

**Authors:** 


**The Thyroid Gland, in Health and Disease.** By ROBERT McGARRISON, M.D., F.R.C.P., Major, Indian Medical Service. Pp. xvii., 2v86. London: Baillière, Tindall & Cox 1917. Price 12s. 6d. net.

This handsome volume, the result of many years of investigation, is divided into three parts, the first dealing with the thyroid and parathyroid glands in health, the second with the factors which cause them to depart from health, and the third with the morbid states—including not only goitre and cretinism, but also tetany, myxœdema, and Graves's disease—which result from this departure.

The great fact which has emerged from the author's investigation is that the most common source of the thyroid's derangement is gastro-intestinal toxæmia.

The author concludes his full account of the treatment of Graves's disease by urging the necessity for more careful examination of every case for foci of infection, and the necessity for their early removal. He is not an advocate for surgical interference, nor does he think that serum treatment possesses any specific action. He advocates hygienic rather than medicinal treatment. The book is one for careful study.

